# Randomized Controlled Trial Comparing 1-Year Outcomes of Low-Energy Femtosecond Laser-Assisted Cataract Surgery versus Conventional Phacoemulsification

**DOI:** 10.3389/fmed.2021.811093

**Published:** 2021-12-17

**Authors:** Yu-Chi Liu, Melina Setiawan, Jia Ying Chin, Benjamin Wu, Hon Shing Ong, Ecosse Lamoureux, Jodhbir S. Mehta

**Affiliations:** ^1^Tissue Engineering and Cell Therapy Group, Singapore Eye Research Institute, Singapore, Singapore; ^2^Cornea and Refractive Surgery Group, Singapore Eye Research Institute, Singapore, Singapore; ^3^Department of Cornea and External Eye Disease, Singapore National Eye Centre, Singapore, Singapore; ^4^Ophthalmology and Visual Sciences Academic Clinical Program, Duke- National University of Singapore (NUS) Medical School, Singapore, Singapore; ^5^Population Health Group, Singapore Eye Research Institute, Singapore, Singapore; ^6^Health Services and System Research Department, Population Health Research, Duke- National University of Singapore (NUS) Medical School, Singapore, Singapore

**Keywords:** low-energy femtosecond laser-assisted cataract surgery, conventional phacoemulsification, randomized controlled trial, aqueous profiles, patient-reported outcomes, clinical outcomes

## Abstract

**Purpose:** To compare 1-year clinical outcomes, phacoemulsification energy, aqueous profiles, and patient-reported outcomes of low-energy femtosecond laser-assisted cataract surgery (FLACS) vs. conventional phacoemulsification.

**Methods:** The study is a randomized controlled trial (RCT) with paired-eye design. Eighty-five patients were randomized to receive FLACS (Ziemer LDV Z8) in one eye and conventional phacoemulsification in the fellow eye. Clinical data including phacoemulsification energy parameters (cumulative dissipated energy, phacoemulsification power, and phacoemulsification time), uncorrected and corrected distance visual acuities (UCDVA and BCDVA), manifest refraction spherical equivalent (MRSE), central corneal thickness (CCT), endothelial cell count (ECC), anterior chamber flare, and post-operative complications were obtained for 1 year. Aqueous humor was collected for the analysis of prostaglandin (PGE)_2_, cytokines and chemokines concentrations. Patients' reported-outcomes on surgical experiences were evaluated using an in-house questionnaire.

**Results:** Compared to conventional phacoemulsification, the low-energy assisted FLACS group had significantly less ECC reduction at 3 months (1.5 ± 0.3% vs. 7.0 ± 2.4%; *P* < 0.01) and 1 year (8.2 ± 2.8% vs. 11.2 ± 3.6%; *P* = 0.03). There were no significant differences in the phacoemulsification energy parameters, UCDVA, BCDVA, MRSE, CCT, occurrence of post-operative complications between the 2 groups throughout post-operative 1 year. Patients' subjective surgical experiences, including the surgical duration and perceived inconvenience, were comparable between the 2 groups. FLACS resulted in significantly higher aqueous PGE_2_ (*P* < 0.01), interleukin (IL)-6 (*P* = 0.03), IL-8 (*P* = 0.03), and interferon (IFN)-γ (*P* = 0.04) concentrations and greater anterior chamber flare at 1 day (*P* = 0.02).

**Conclusions:** Our RCT presented 1-year longitudinal clinical and laboratory data. The long-term ECC result was more favorable in low-energy FLACS. The rest of the intraoperative and post-operative outcomes, as well as patient-reported outcomes, were comparable between these two procedures.

## Introduction

Femtosecond laser-assisted cataract surgery (FLACS) has been shown to be a safe and effective procedure (1), and increasingly being incorporated into surgical practice. Since its introduction in 2010 (2), numerous studies have been conducted to compare the clinical outcomes with those of conventional phacoemulsification. These results, however, were mainly from observational cohort studies and were not randomized controlled trials. Current published RCTs reported only limited post-operative outcomes, and the duration of follow-up was short (mostly 3 months) (3–8). Long-term RCTs with a paired-eye design, which is the most valid way of comparing FLACS and conventional phacoemulsification, are currently limited. Furthermore, literature on patient-reported outcomes mainly focus on the visual quality or quality of life following FLACS, but reports on surgical experiences in FLACS are lacking. As a concern of FLACS is the high cost, understanding patients' subjective surgical experiences, on top of visual quality, may provide another perspective for surgeons' consideration when deciding the choice of surgical procedure.

Among the five commercially available laser platforms, the Femto LDV Z8 (Ziemer Ophthalmic Systems AG, Port, Switzerland) delivers the laser spots in small spot size, and has a high numerical aperture as well as a low energy range [nanojoules (nJ) per pulse] with high frequency (9, 10). These characteristics may provide advantages of better precision of the laser cutting and reduction in the extent of collateral tissue damage (11). In addition, as the Femto LDV Z8 laser system is mobile and has the smallest footprint compared to the other femtosecond laser platforms, the procedure can be completed in the same operating table. This thus effectively overcomes the logistic difficulties in patient transfer, which is encountered with other laser systems and can slow down the patient flow. Studies on the clinical outcomes of low-energy FLACS are limited, and they have been limited to short-term reports or with limited clinical assessments (12). Three-months post-operative changes of central corneal thickness (CCT), endothelial cell count (ECC), and aqueous flare levels were reported when the low-energy system was introduced (12). However, a comparative conventional surgery arm was not included in that study. In another study, Pajic et al. presented that the 3-months visual and refractive outcomes in low-energy FLACS were comparable to those in conventional phacoemulsification (13). However, no other clinical parameters were further assessed.

It has been shown that the aqueous prostaglandin (PGE) level was significantly higher in FLACS than conventional phacoemulsification (1, 14, 15), and this PGE rise is a causative factor for intraoperative miosis (14, 16). Unlike the findings in many FLACS studies using high-energy systems in which significant intraoperative miosis was noted (17–20), two recent studies reported that there were no statistically significant changes between pre-operative and post-laser pupil areas following low-energy FLACS (21, 22). This may imply that the low-energy system offers the advantages of inducing less tissue reaction and less resultant PGE_2_ release. Analysis of aqueous humor would allow us to understand more about how the low-energy system affects the breakdown of blood-aqueous barrier at a molecular level.

Phacoemulsification energy utilized during the surgery is another parameter that affects post-operative outcomes. Studies comparing phacoemulsification energy parameters in FLACS to conventional phacoemulsification have demonstrated inconsistent results, although the majority of the literature showed a significant difference in favor of FLACS, with respect to the cumulative dissipated energy (CDE) and phacoemulsification time (23). Whether the low-energy system provides greater benefits in the energy profile and whether this could be a beneficial option for patients with low endothelial cell density or dense cataract, has not been studied.

In the present study, we aimed to conduct a RCT with a paired-eye design to compare low-energy FLACS and conventional phacoemulsification in the same patient, with the main advantage of being that the outcomes were assessed with the elimination of inter-subject bias. The comprehensive data, covering 1-year clinical outcomes, aqueous humor profiles, phacoemulsification energy, and patient-reported outcomes on surgical experiences, were collected and compared.

## Methods

### Study Designs and Patients

This study was a registered RCT (NCT03351894) in which we recruited 85 patients with bilateral cataracts from December 2017 to November 2019. The inclusion and exclusion criteria are listed in Supplementary Table 1. Approval for the study was granted by the institutional review board of SingHealth, Singapore (Number: 2015/2565), and the study was conducted in accordance to the Declaration of Helsinki. The randomization was performed using random allocation cards from computer-generated random numbers and allocated patients to each treatment group. Each patient underwent either conventional phacoemulsification or FLACS in the right eye, followed by FLACS or conventional surgery in the left eye, which was operated 3–4 weeks apart from the surgery of the right eye.

### FLACS and Phacoemulsification Procedure

All patients were prescribed 0.5% preservative-free cyclopentolate hydrochloride and 0.5% levofloxacin eye drops four times daily 1 day before surgery. Mydriasis was maintained with 0.5% tropicamide and 2.5% phenylepherine hydrochloride eye drops instilled three times within 1 h prior to surgery. All procedures were performed under local anesthesia with sedation.

The FLACS procedure was performed with the LDV Z8 system. The suction interface was filled up with balanced salt solution to create a fluid-patient interface. The hand-piece of the articulating arm was docked on the interface angled at −10° over (10). Laser pre-treatment started with an anterior capsulotomy with a pre-set diameter of 5.0 mm at 90% energy, followed by lens fragmentation with a 6-sector pie-cut pattern at 100% energy, and then a 2.6 mm corneal incision. Within 5 min of the completion of laser pre-treatment, ~150 μL of aqueous humor was collected through a limbal paracentesis using a 30-gauge needle. Standard phacoemulsification and intraocular lens insertion were then performed. For the conventional phacoemulsification group, aqueous humor was collected in an identical way as that for the FLACS group. All aqueous samples were immediately transferred on dry ice to a laboratory, and the supernatants were stored at −80°C until analysis. The surgery, in both FLACS and conventional phacoemulsification groups, was performed with the same phacoemulsification machine (Infiniti Vision Ozil system, Alcon Laboratories, Inc., Fort, Worth, TX), with the same model of intraocular lens implantation (SA60AT, Alcon). The surgery was performed by 2 consultant-grade cataract surgeons (J.S.M and H.S.O). At the end of the surgery, the CDE, phacoemulsification power, and phacoemulsification time were recorded. All patients were given 0.5% levofloxacin and preservative-free dexamethasone eye drops 3 h for a week starting from the next day of surgery, and then tapered until 2 times daily over 4 weeks.

The pupil diameter and area were measured before and after laser treatment for the FLACS group, using the images with the same magnification captured from surgical videos and ImageJ software. The actual pupil area was calculated by using the following proportional formula: Actual pupil area (mm^2^) = (Video pupil area)/(Video capsulotomy area) × π (Set capsulotomy diameter/2)^2^.

### Clinical Evaluation and Patient-Reported Outcomes on Surgical Experiences

Patient data collected included patient's age, gender, lens density assessed using the software Pentacam Nucleus Staging (PNS), uncorrected and best-corrected distance visual acuities (UCDVA and BCDVA) in logarithm of the minimum angle of resolution (logMAR) values, manifest refraction spherical equivalent (MRSE), CCT (Visante, Carl Zeiss, Dublin CA, USA), ECC (EB-10 specular microscopy, Konan Medical, Inc., Irvine, CA), and aqueous flare levels (flare meter, FM-600, Kowa, CA, USA). These assessments were performed at different study time points (Supplementary Table 2) over the study period of 1 year. For the ECC measurement, the central ECC was measured for 3 times using a fixed-frame method of cell counting, marking at least 100 cells per image by an experienced technician (24). The average was used for statistical analysis. Intraoperative and post-operative complications were also recorded. Patients' subjective pre-, peri-, and post-surgical experiences were evaluated 1 day after surgery by using an in-house 7-item questionnaire on a 10-point scale. The items included nervousness and confidence in the procedure, intraoperative discomfort, post-operative pain, visual satisfaction, and subjective feeling about the surgical duration and inconvenience.

### Aqueous Analysis

The concentration of PGE_2_ was analyzed using enzyme-linked immunosorbent assay (ELISA) kits (Cayman Chemical Co.). An immunoassay kit (Procartaplex Human Cytokine/Chemokine/Growth Factor Panel 1, Thermo Fisher Scientific, Inc.) was used to measure 45 cytokines, chemokines, and growth factors.

### Statistical Analysis

The sample size was calculated using the results of the first six patients and CDE as the primary outcome, with a paired-eye design, power of 80%, significance level of 5%, and non-inferiority margin of 10%. The sample size of 77 patients was required to confirm the differences in the CDE between the FLACS and conventional phacoemulsification groups (CDE = 20.2 ± 5.8 and 22.8 ± 5.6 s, respectively, for the first 6 patients). Considering a 10% lost follow-up rate, we therefore recruited 85 patients. A paired-*t* test was used to compare the values between the FLACS and conventional phacoemulsification groups. Repeated–measures ANOVA and *post-hoc* tests were used to analyze the data of different follow-up visits. The correlation between the aqueous PGE_2_ level and pupil area, as well as between the aqueous cytokines/chemokines/growth factors and post-operative aqueous flare level, was assessed with a Pearson correlation test. Each item of the patients' reported outcome scale was assessed individually. All data were expressed as mean ± standard deviation, and *P*-values < 0.05 were considered statistically significant (STATA; STATACrop, College Station, TX).

## Results

The mean patient age was 69.5 ± 6.8 years (female: male = 37:48). There were 4 patients who were lost the follow up during the 1-year study period due to COVID-19 pandemic (CONSORT diagram; Supplementary Figure 1).

### Clinical Outcomes

The PNS grade was comparable between the FLACS (1.9 ± 1.0) and conventional phacoemulsification (2.0 ± 0.9) groups (*P* = 0.88), and there were no differences in the phacoemulsification energy parameters between 2 procedures. The mean CDE was 20.3 ± 6.7 and 21.2 ± 6.0 s (*P* = 0.82), phacoemulsification power was 30.1 ± 9.2% and 31.8 ± 10.5% (*P* = 0.63), and phacoemulsification time was 8.2 ± 3.0 and 9.3 ± 3.3 s (*P* = 0.47), for the FLACS and conventional procedures, respectively. For the FLACS group, the mean pupil area significantly reduced after laser pre-treatment, from 40.5 ± 8.9 to 32.5 ± 10.4 mm^2^ (*P* = 0.03).

There were no intraoperative complications. Clinically cystoid macular edema (CME) was observed in 2 eyes in the conventional group (2.4%) and in 1 eye (1.2%) in the FLACS group (*P* = 0.56), occurring during 1–3 months post-operatively. As CME affected the BCDVA and UCDVA assessments, and the treatment with topical non-steroidal anti-inflammatory drug (NSAID) interfered with the aqueous flare level, these 3 subjects were excluded from the statistical analysis.

**Figure 1:**
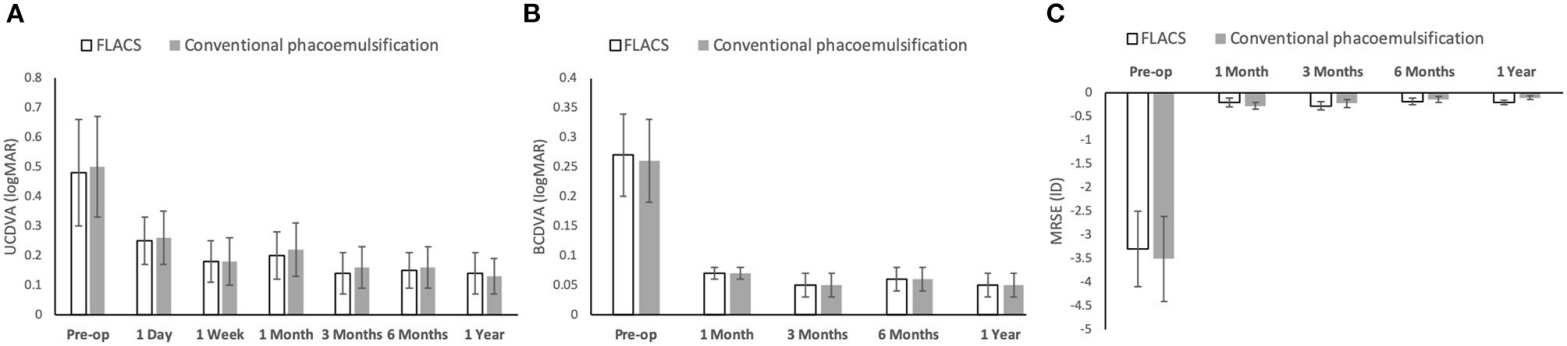
Bar graph showing the visual and refractive outcomes following FLACS and conventional phacoemulsification over the study period of 1 year. There was no significant difference in the UCDVA **(A)**, BCDVA **(B)**, and MRSE **(C)** between the two groups at all the post-operative time points. Error bars indicate standard deviation.

The pre-operative mean BCDVA and MRSE were comparable between the 2 groups (*P* = 0.69 and *P* = 0.56). We did not observe significant differences in the mean post-operative UCDVA, BCDVA and MRSE at all time points (Figures 1A–C; *P* > 0.05 for all time points). The BCDVA at 1 year was 0.05 ± 0.01 logMAR and 0.05 ± 0.01 logMAR, and the MRSE at 1 year was −0.20 ± 0.05 and −0.10 ± 0.04 diopters (D) for the FLACS and conventional groups (*P* = 0.91 and *P* = 0.33, respectively). Five and four eyes developed mild posterior capsule opacification (PCO) in the FLACS and conventional groups, respectively (6.4 and 5.1%; *P* = 0.73).

**Figure 2:**
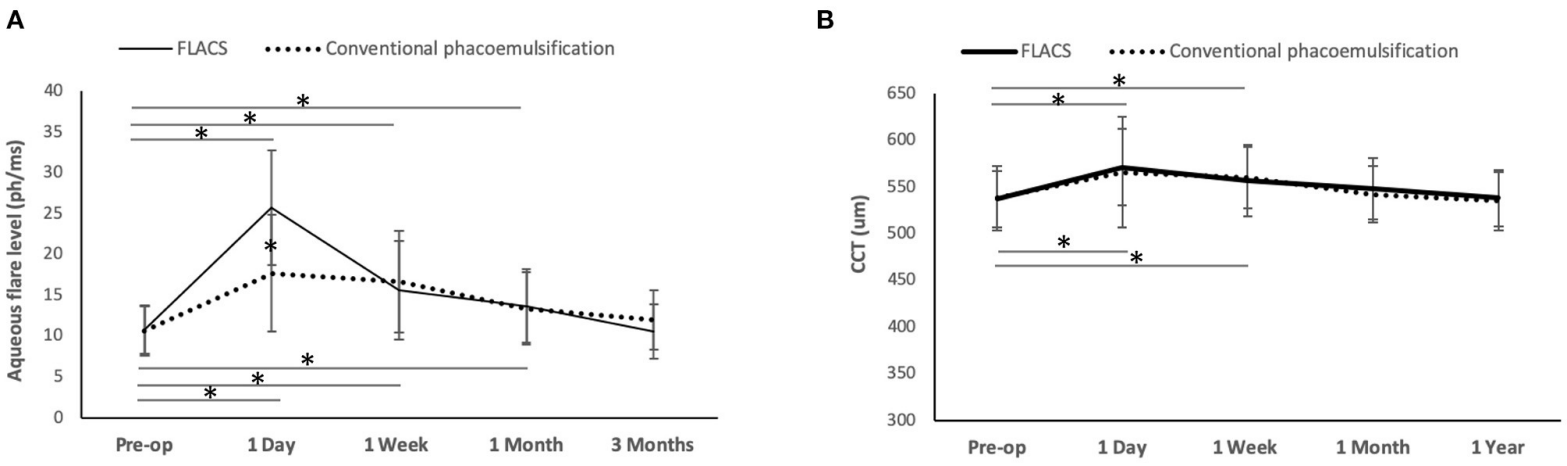
Line graph showing the changes of aqueous flare levels and CCT after FLACS and conventional phacoemulsification. The flare levels significantly increased for one month post-operatively in both groups, and eyes underwent FLACS had significantly greater aqueous flare level than those underwent conventional phacoemulsification at day 1 **(A)**. The CCT increased significantly at 1 day and 1 week, regardless of the group. There was no significant difference between the two groups in the CCT at all the time points **(B)**. Error bars indicate standard deviation. *P < 0.05.

A significant increase of aqueous flare levels was observed after surgery for 1 month in both FLACS and conventional groups (day 1: *P* < 0.001 and *P* < 0.001; week 1: *P* = 0.018 and *P* = 0.023; month 1: *P* = 0.039 and *P* = 0.028 for the FLACS and conventional groups, respectively, when comparing to pre-operative levels). Eyes with FLACS treatment had significantly greater aqueous flare levels than eyes with conventional surgery at day 1 (25.7 ± 11.4 vs. 17.7 ± 9.2 ph/ms; *P* = 0.02), but no significant difference was noted thereafter (Figure 2A).

The CCT significantly increased for 1 week post-operatively regardless of groups (day 1: *P* = 0.011 and *P* = 0.016; week 1: *P* = 0.029 and *P* = 0.026 for the FLACS and conventional phacoemulsification groups, respectively, when comparing to pre-operative values). The difference in the CCT between the 2 groups was not significant throughout the study period of 1 year (Figure 2B). The absolute ECC value was comparable between the 2 groups at all time points. However, when evaluating the percentage of changes in ECC after surgery, the conventional group had significant ECC reduction at 1 year (11.2 ± 3.6% decrease; *P* = 0.012 when comparing to the pre-operative level), while the ECC change was not statistically significant in the FLACS group (8.2 ± 2.8% decrease; *P* = 0.08 when comparing to the pre-operative level). At 3 months and 1 year, eyes that underwent FLACS presented with significantly less post-operative ECC changes than eyes that underwent conventional phacoemulsification (*P* < 0.01 and *P* = 0.03, respectively; Table 1).

**Table 1:** Post-operative ECC changes in 2 groups over 1-year period.

**ECC (% of decrease from pre-op level)**	**Pre-op**	**3 Months**	**6 Months**	**1 Year**
FLACS	2,622 ± 315	2,583 ± 624 (1.5 ± 0.3%)	2,434 ± 448 (7.2 ± 1.9%)	2,406 ± 486 (8.2 ± 2.8%)
Conventional phacoemulsification	2,649 ± 419	2,462 ± 589 (7.0 ± 2.4%)	2,433 ± 532 (8.2 ± 2.6%)	2,353 ±4 16 (11.2 ± 3.6%)
*P-*value^*^		**<0.01**	0.26	**0.03**

* *Comparison of the % of decrease between 2 groups*.

*Bold values mean significant P values*.

For patient-reported outcomes on surgical experiences, no significant difference was shown between the 2 groups for the post-operative pain, confidence in the procedure, visual satisfaction, nervousness, and subjective feeling about the surgical duration and inconvenience, although the discomfort experienced during the surgery was borderline significantly greater in the FLACS than in the conventional group (*P* = 0.05; Table 2).

**Table 2:** Scores of the patients' subjective pre-, peri-, and post-surgical experiences in the two groups.

**Question**	**FLACS**	**Conventional phacoemulsification**	***P-*value**
How nervous were you for the surgery? (1: least; 10: most)	3.1 ± 1.8	2.5 ± 1.5	0.34
How much discomfort did you experience during the surgery? (1: least; 10: most)	2.1 ± 0.8	1.2 ± 0.7	0.05
How much confidence did you have in the surgery? (1: least; 10: most)	8.5 ± 1.4	8.6 ± 1.5	0.85
How long did you feel the surgery take? (1: quickest; 10: longest)	7.0 ± 2.2	6.7 ± 1.8	0.46
How much pain did you experience after the surgery? (1: least; 10: most)	1.9 ± 0.9	1.5 ± 0.7	0.37
How satisfied are you with the visual outcome after the surgery? (1: least; 10: most)	8.0 ± 1.4	8.4 ± 1.2	0.13
Please rate the convenience or inconvenience of the overall surgical procedure? (0: inconvenient; 10:convenient)	8.1 ± 1.8	8.5 ± 1.7	0.23

### Aqueous Analysis

The FLACS group had a significantly higher aqueous PGE_2_ level than conventional phacoemulsification (62.2 ± 22.4 vs. 23.8 ± 11.7 pg/mL; *P* < 0.01). FLACS also resulted in significantly higher aqueous interleukin (IL)-6 (13.6 ± 3.8 vs. 5.5 ± 2.8 pg/mL; *P* = 0.03), IL-8 (13.4 ± 4.8 vs. 5.5 ± 1.6 pg/mL; *P* = 0.03), and interferon (IFN)-γ (6.8 ± 1.9 vs. 0.3 ± 0.1 pg/mL; *P* = 0.04) concentrations. There was no significant difference between the 2 groups for the rest of the analytes (Table 3). The percentages of change in pupil area had moderate, significant and negative correlation with the aqueous PGE_2_ levels (*r* = −0.61; *P* = 0.02). We did not observe significant correlation between the significantly increased analytes (PGE_2_, IL-6, IL-8, and IFN-γ) and anterior chamber flare level at all time points (all *P* > 0.05).

**Table 3:** Aqueous humor concentrations of cytokines, chemokines and growth factors.

	**Conventional phacoemulsification**	**FLACS**	***P*-value**
***Cytokines*** **(pg/mL)**			
IL-1 α	0.1, 0.06	1.1, 0.8	0.48
IL-1 β	0.8, 0.5	0.8, 0.4	0.82
IL-1RA	532.4, 299.8	988.3, 684.3	0.36
IL-4	0.2, 0.2	0.9, 0.7	0.33
IL-5	1.4, 0.8	1.8, 1.0	0.62
IL-6	5.5, 2.8	13.6, 3.8	**0.03**
IL-7	325.7, 112.1	333.6, 172.7	0.78
IL-8	5.5, 1.6	13.4, 4.8	**0.03**
IL-9	2.5, 1.9	12.5, 10.2	0.26
IL-10	0.04, 0.02	0.07, 0.04	0.60
IL-18	1.8, 0.8	2.0, 0.9	0.76
IL-21	3.4, 2.8	10.0, 8.2	0.27
IL-22	96.6, 55.8	109.6, 76.1	0.66
IL-23	0.8, 0.5	8.2, 5.5	0.10
IL-27	10.5, 6.4	12.8, 8.5	0.79
IL-31	11.9, 7.9	16.0, 10.5	0.31
IFN-α	0.3, 0.2	0.4, 0.2	0.87
IFN-γ	0.3, 0.1	6.8, 1.9	**0.04**
LIF	6.0, 3.8	6.9, 4.5	0.79
BDNF	0.9, 0.3	0.7, 0.3	0.88
TNF-α	0.2, 0.2	0.8, 0.5	0.79
***Chemokines*** **(pg/mL)**			
Eotaxin	4.5, 2.5	4.2, 2.0	0.82
SCF	4.9, 3.0	5.5, 2.8	0.77
GRO-α	15.5, 9.9	18.6, 12.8	0.46
IP-10	234.6, 142.7	356.5, 246.6	0.32
MIP-α	35.2, 28.1	33.0, 25.0	0.83
MIP-1β	52.6, 30.1	55.3, 29.3	0.62
MCP-1	3, 755.9, 1, 265.3	4, 131.6, 2, 521.8	0.44
RANTES	36.9, 22.0	42.7, 25.8	0.50
SDF-α	1, 622.8, 993.4	1, 823.5, 1, 043.2	0.39
***Growth factors*** **(pg/mL)**			
EGF	1.1, 0.6	2.0, 1.4	0.39
VEGF-α	1, 572.7, 692.4	1, 625.3, 1, 043.8	0.68
VEGF-D	0.4, 0.3	0.5, 0.3	0.90
FGF-2	1, 896.9, 1, 043.7	1, 922.4, 943.7	0.45
HGF	432.8, 289.1	475.3, 175.9	0.22
PIGF-1	5.5, 1.8	7.9, 3.2	0.20
PDGF-BB	17.5, 7.0	25.8, 16.1	0.41

*SCF, stem cell factor; GRO, growth-regulated oncogene; IP, interferon-inducible protein; MCP, monocyte chemoattractant protein; SDF, stromal cell-derived factor; HGF, hepatocyte growth factor; PIGF, placental growth factor*.

*Bold values mean significant P values*.

## Discussion

Through a paired-eye RCT, we present comprehensive data on 1-year outcomes, including clinical results, phacoemulsification energy data, aqueous profiles, and patient-reported outcomes, in low-energy FLACS in comparison with conventional phacoemulsification. The strength of this study is the randomized trial design, as well as the use of data of paired eyes from the same patient, providing a more accurate assessment by minimizing inter-eye and inter-individual variations as well as selection bias. The visual, refractive and patient-reported outcomes on surgical experiences were comparable between these two procedures, although FLACS was associated with significantly higher degree of anterior chamber inflammation on the first day post-operatively. The ECC loss at 1 year was significantly less in low-energy FLACS. Similar to other FLACS laser platforms, there was significant release of PGE_2_ and several pro-inflammatory cytokines such as IL-6, IL-8 and IFN-γ, in the aqueous.

The post-operative CCT significantly increased for 1 week regardless of the groups, with no significant differences between the 2 groups in the CCT and absolute ECC values throughout the 1-year study period. But it was observed that the FLACS group had significantly less ECC loss than conventional surgery at 1 year (8.2 vs. 11.2%). This finding is consistent with a retrospective observational study showing that the ECC loss was 12.4% and 18.1% for the low-energy FLACS vs. conventional bimanual microincision cataract surgery at 18 months post-operatively (25). The more favorable impact of FLACS on post-operative ECC change has been reported in the literature regardless of the femtosecond laser system used (1, 26), suggesting the protective effects of FLACS for those who are susceptible to intraoperative and post-operative ECC loss. Future studies may include the comparisons of ECC changes between low-energy and high-energy systems to elucidate this further. With respect to visual and refractive outcomes, meta-analyses have demonstrated that these two procedures are equivalent (1, 27), and this was also seen in the present study.

Studies comparing phacoemulsification energy parameters in high-energy FLACS vs. conventional phacoemulsification have demonstrated inconsistent results. The heterogeneous results in the energy profiles (CDE and phacoemulsification time) in previously published studies may result from the disparity in the patient selection, laser platform used, surgical techniques, and the calculation formula used in different phacoemulsification machines (1, 28). However, a recent systematic review showed significant differences in favor of FLACS in terms of the CDE and effective phacoemulsification time (23). At present, there is only one study published on the comparison of phacoemulsification energy between the low-energy FLACS and conventional surgery, and the authors reported significantly lower effective phacoemulsification time in FLACS (13). We did not observe such differences in the CDE, phacoemulsification power and phacoemulsification time in our study, and it may be due to several reasons. Firstly, the above-mentioned study was not conducted in a contralateral eye design, and the variability in the cataract density in different individuals recruited in 2 groups might have introduced bias. Secondly, the cataract severity in our cohort was relatively mild (mean PNS grade = 1.9 and 2.0 for the FLACS and conventional groups). Ang et al. evaluated the differences in the CDE in FLACS vs. conventional phacoemulsification stratified by the Lens Opacities Classification System III grading, and significant differences in the CDE were only seen for patients with nuclear opalescence grade 4, but not for those with less than grade 4 opalescence (26). Thirdly, the majority of the surgery in the present study was performed by a senior consultant with 30-years of experience. With skillful phacoemulsification techniques, the advantages FLACS provides in ultrasound energy might be mitigated.

Unlike other studies that focused on the visual quality and quality of life in the patient-reported outcomes assessment, our study focuses on patient-reported surgical experiences. The questionnaire was conducted on the next day after surgery to avoid recall bias. Besides objective visual outcomes, the self-reported visual outcome was also comparable between these two types of surgery (Table 2). Of note, there was no difference in the subjective feelings with respect to post-operative pain, surgical time, inconvenience, and confidence in the surgical procedure. The perception of surgical duration is an important parameter affecting patient post-operative satisfaction. Unlike other FLACS procedures in which patients have to be transferred to another room following the laser procedure, the Ziemer LDV is a mobile system with a small footprint that allows the surgeon to push away the laser arm and complete the surgery on the same operating table. These might explain the high convenience score in the FLACS group, and the comparable score in the surgical time in the two groups. Patients reported borderline significantly greater intraoperative discomfort when receiving FLACS, and this might come from the docking, suction and laser pre-treatment steps, which lasted for ~3.2 min. However, the discomfort score was low for both of the procedures (2.1 ± 0.8 vs. 1.2 ± 0.7 for the FLACS and conventional phacoemulsification groups, respectively).

Significant reduction in the pupil area was observed in the present study, while unchanged pupil size was reported in two previous studies in which the low-energy system and no pre-operative NSAID were used (21, 22). However, those studies were conducted with fewer patient numbers and might be underpowered. Moreover, our FLACS procedure included the creation of a corneal incision, which might also result in a PGE_2_ increase. The rise in PGE_2_ in FLACS has been reported to cause intraoperative miosis (14, 16, 29), and the use of pre-operative NSAID therefore has been suggested to reduce the extent and occurrence of intraoperative miosis (14, 20). Pre-operative NSAID was not prescribed in this study, as the study was initiated in 2017 when the concept of the use of NSAID in FLACS was not fully recognized. Of note, when comparing the PGE_2_ level of our FLACS group with those of published studies in which a high-energy (μJ) platform and no pre-operative NSAID were used, the aqueous PGE_2_ concentration was much lower with the use of the nJ-system (62.2 pg/mL vs. 377.1 to 1,911.4 pg/mL) (16, 20, 30, 31). The PGE_2_ concentration reported in the present study was also lower than those reported in the studies conducted with high-energy systems and with the use of pre-operative NSAID (65.3–743.6 pg/mL) (20, 30, 31). This highlights the potential advantage of the low-energy system as the result of fewer cavitation bubbles generated during lens fragmentation and minimal collateral tissue damage on the unpigmented epithelial cell layer of the ciliary body, triggering less PGE_2_ release (14, 32, 33). In our study, the correlation between the aqueous PGE_2_ level and the percentage of change in pupil area was only moderate, indicating that in addition to PGE_2_, other factors, such as cholinergic or antisympathetic pathway, or anatomic dynamics of the pupil (34, 35), also play a role. The release of PGE_2_ intraoperatively is also a proposed etiological factor for post-operative CME (36). Meta-analysis data have shown that there was no significant difference in the incidence of CME between FLACS and conventional phacoemulsification (23), which is in alignment with our study. Nuffel et al. further evaluated the changes in the central subfield macular thickness in low-energy FLACS vs. conventional surgery and reported no statistically significant difference (37).

Even with the low energy per spot, significantly greater release of pro-inflammatory and inflammatory cytokines, such as IL-6, IL-8 and IFN-γ, was still observed in the aqueous in the FLACS group. This might result from the breakdown of blood-aqueous barrier because of the shockwave, vibrations and temperature increase when laser spots passed through the aqueous humor (33). The increased cytokines could also account for the significantly greater anterior chamber flare values in the FLACS group at post-operative day 1, as IL-6, IL-8, and IFN-γ are known pro-inflammatory cytokines (38–40). IL-6 has been shown to play a role in the development of PCO after cataract surgery (41, 42). However, we did not observe a significant difference in the incidence of PCO between the two groups. Evaluation of PCO with a more detailed PCO grading system to quantify the opacification severity may help to distinguish more in future studies. Of note, the aqueous IL-6 level in the present study (13.6 pg/mL) was lower than those reported in the literature where high-energy FLACS platforms were used, with or without NSAID (24.6–57.6 pg/mL) (15, 43). The lack of significant correlation between significantly increased analytes (PGE_2_, IL-6, IL-8, and IFN-γ) and anterior chamber flare level suggests that other cytokines, in conjunction with the cellular components of inflammatory cells, collectively account for the anterior chamber reaction.

In summary, the strength of our study is the RCT with paired-eye design and relatively low lost follow-up rate (4.7%), allowing us to achieve robust data collection and comparison over 1 year. We demonstrated for the first time the comparisons of low-energy FLACS vs. conventional phacoemulsification, with respect to clinical results, phacoemulsification energy, aqueous cytokine/chemokine profiles, and patient-reported outcomes. The visual, refractive and patient-reported outcomes on surgical experiences, phacoemulsification energy, post-operative CCT, and the occurrence of CME and PCO were comparable between these two procedures. The post-operative ECC loss was in favor of low-energy FLACS. Despite low energy per laser spot, patients receiving FLACS had significantly higher aqueous PGE_2_, IL-6, IL-8, and IFN-γ levels, leading to greater anterior chamber inflammation on the 1st day after surgery. Nevertheless, the increased aqueous PGE_2_ and IL-6 levels were lower than those in published studies with high-energy systems. Our study findings expand the knowledge on FLACS, with a comprehensive presentation ranging from objective clinical measures and subjective patient-reported outcomes, to laboratory analysis.

## Data Availability Statement

The raw data supporting the conclusions of this article will be made available by the authors, without undue reservation.

## Ethics Statement

The studies involving human participants were reviewed and approved by the Institutional Review Board of SingHealth, Singapore (Number: 2015/2565). The patients/participants provided their written informed consent to participate in this study.

## Author Contributions

Y-CL collected the data, performed the analyses, and wrote the manuscript. MS, JC, and BW performed the laboratory analysis. HO collected the data and revised the manuscript. EL and JM supervised the manuscript writing. All authors contributed to the article and approved the submitted version.

## Funding

This research was supported by the Singapore National Eye Center HREF Grant (R1249/55/2015).

## Conflict of Interest

The authors declare that the research was conducted in the absence of any commercial or financial relationships that could be construed as a potential conflict of interest.

## Publisher's Note

All claims expressed in this article are solely those of the authors and do not necessarily represent those of their affiliated organizations, or those of the publisher, the editors and the reviewers. Any product that may be evaluated in this article, or claim that may be made by its manufacturer, is not guaranteed or endorsed by the publisher.
