# Supplementary material for: Randomized Controlled Trial Comparing 1-Year Outcomes of Low-Energy Femtosecond Laser-Assisted Cataract Surgery versus Conventional Phacoemulsification
Source: Front Med (Lausanne). 2021 Dec 17;8:811093. doi: 10.3389/fmed.2021.811093 (PMC8718704; doi:10.3389/fmed.2021.811093)
Supplement: Supplementary file 1 [file Data_Sheet_1.docx]

**Supplementary Table 1. Inclusion and exclusion criteria for study participants**

| ***Inclusion criteria*** |
| --- |
| Adult aged > 55 years with visually symptomatic senile cataract in both eyes |
| Similar cataract severity between eyes, with PNS difference less than 2 |
| Targeted refractive power after surgery within ± 0.5 dioptre of emmetropia |
| Patients who are willing and able to return for scheduled follow-up examinations for 1 year after surgery |
| Patients have medically dilated pupil size of at least 5.0 mm |
| ***Exclusion criteria*** |
| Any corneal abnormalities, such as significant corneal asymmetry or irregular topography, corneal opacity, corneal scar, previous corneal or refractive surgery |
| Eyes with signs of zonular dialysis or phacodonesis |
| Eyes with signs of lens dislocation or subluxation |
| Patients scheduled to undergo combined surgery such as cataract surgery and corneal transplantation |
| Patients with other ocular comorbidity such as retinal diseases, glaucoma, optic nerve diseases, that may affect the outcome measures |
| Patients who were taking oral NSAID |

**Supplementary Table 2. Clinical assessments over 1-year study period**

|  | Pre-op | 1 day | 1 week | 1 month | 3 months | 6 months | 1 year |
| --- | --- | --- | --- | --- | --- | --- | --- |
| UCDVA | * | * | * | * | * | * | * |
| BCDVA | * | * | * | * | * | * | * |
| MRSE | * | * | * | * | * | * | * |
| Flare meter | * | * | * | * | * |  |  |
| CCT | * | * | * | * |  |  | * |
| ECC | * |  |  |  | * | * | * |

UCDVA: uncorrected distance visual acuity; BCDVA: best corrected distance visual acuity; MRSE: manifest refraction spherical equivalent; CCT: central corneal thickness; ECC: endothelial cell count

**Supplementary Figure Legends:**

**Supplementary Figure 1**. CONSORT flow diagram illustrating enrollment, allocation, follow-up and analysis.
