# Supplementary figures and images for: Randomized Controlled Trial Comparing 1-Year Outcomes of Low-Energy Femtosecond Laser-Assisted Cataract Surgery versus Conventional Phacoemulsification
Source: Front Med (Lausanne). 2021 Dec 17;8:811093. doi: 10.3389/fmed.2021.811093 (PMC8718704; doi:10.3389/fmed.2021.811093)

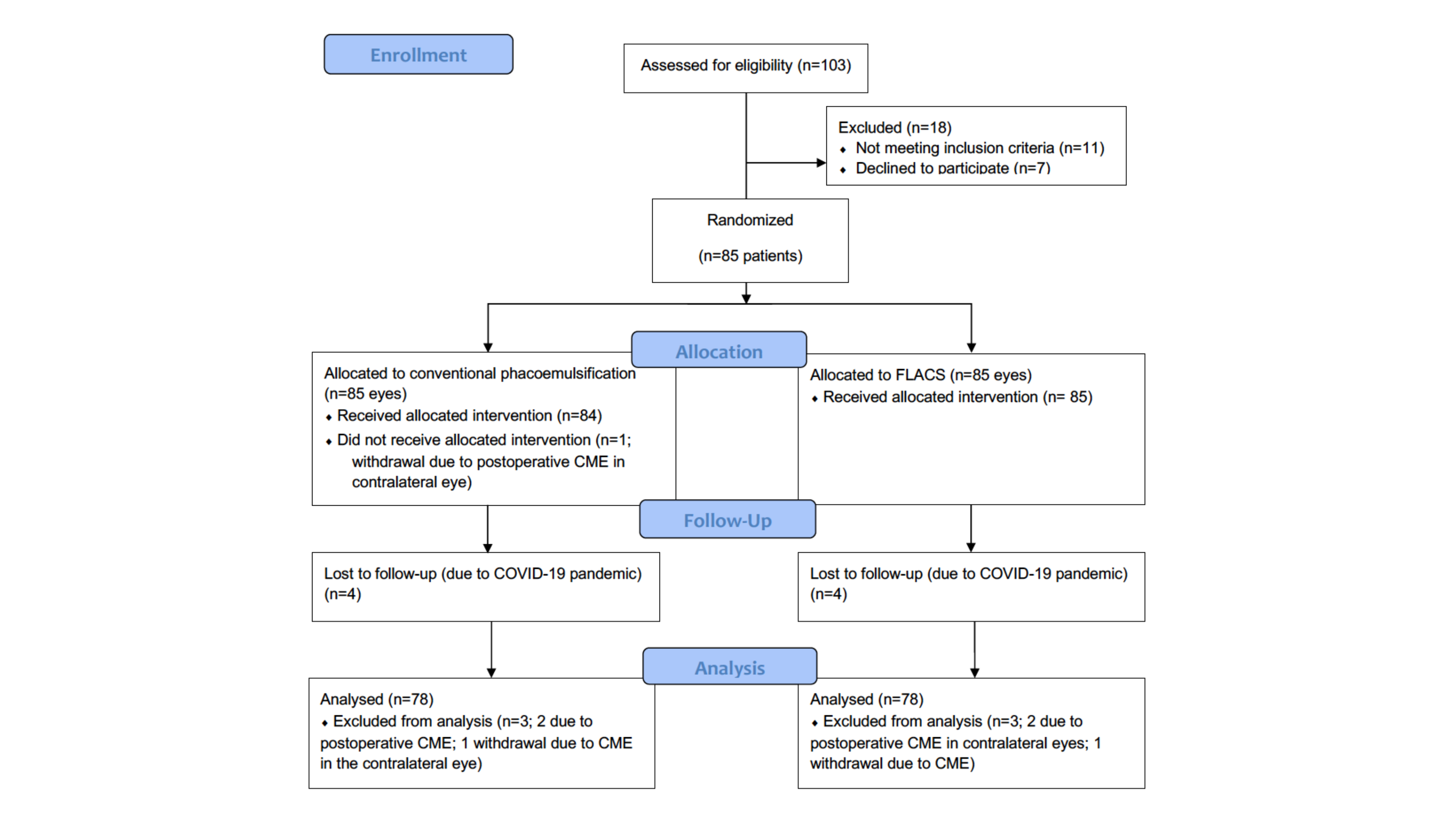

Supplement: Supplementary file 2 [file Image_1.TIF]
